# G protein-coupled receptor 35 stimulation reduces osteoclast activity in primary human bone cells

**DOI:** 10.1093/jbmrpl/ziaf131

**Published:** 2025-08-06

**Authors:** Maria L Price, Rachael A Wyatt, Ana Crastin, Aqfan Jamaluddin, Rowan S Hardy, Morten Frost, Caroline M Gorvin

**Affiliations:** Department of Metabolism and Systems Science and Centre for Diabetes, Endocrinology and Metabolism (CEDAM), University of Birmingham, Birmingham B15 2TT, United Kingdom; Centre for Membrane Proteins and Receptors (COMPARE), Universities of Birmingham and Nottingham, Birmingham B15 2TT, United Kingdom; Department of Metabolism and Systems Science and Centre for Diabetes, Endocrinology and Metabolism (CEDAM), University of Birmingham, Birmingham B15 2TT, United Kingdom; Centre for Membrane Proteins and Receptors (COMPARE), Universities of Birmingham and Nottingham, Birmingham B15 2TT, United Kingdom; Department of Metabolism and Systems Science and Centre for Diabetes, Endocrinology and Metabolism (CEDAM), University of Birmingham, Birmingham B15 2TT, United Kingdom; National Institute for Health and Care Research (NIHR) Biomedical Research Centre (BRC), University of Birmingham, B15 2TT, United Kingdom; Department of Metabolism and Systems Science and Centre for Diabetes, Endocrinology and Metabolism (CEDAM), University of Birmingham, Birmingham B15 2TT, United Kingdom; Centre for Membrane Proteins and Receptors (COMPARE), Universities of Birmingham and Nottingham, Birmingham B15 2TT, United Kingdom; Department of Metabolism and Systems Science and Centre for Diabetes, Endocrinology and Metabolism (CEDAM), University of Birmingham, Birmingham B15 2TT, United Kingdom; Department of Biomedical Sciences, University of Birmingham, Birmingham B15 2TT, United Kingdom; Molecular Endocrinology Laboratory (KMEB), Department of Endocrinology, Odense University Hospital, DK-5000, Odense C, Denmark; Department of Clinical Research, Faculty of Health Sciences, University of Southern Denmark, DK-5000, Odense C, Denmark; Department of Metabolism and Systems Science and Centre for Diabetes, Endocrinology and Metabolism (CEDAM), University of Birmingham, Birmingham B15 2TT, United Kingdom; Centre for Membrane Proteins and Receptors (COMPARE), Universities of Birmingham and Nottingham, Birmingham B15 2TT, United Kingdom

**Keywords:** bone resorption, G protein signaling, orphan GPCR, osteoporosis, TRAP

## Abstract

G protein-coupled receptor 35 (GPR35) is an orphan receptor that is widely expressed in tissues, including human osteoblasts and osteoclasts. Expression of the GPR35 gene and protein is downregulated in osteoporosis patients and in mouse models of the disease. *Gpr35*-knockout mice have reduced bone mass, while GPR35 agonism rescues bone loss in rodent osteoporosis models, indicating that GPR35 has an important role in bone. Our previous studies demonstrated *GPR35* is expressed in human osteoclasts, and we sought to determine the receptors function in these cells. We differentiated human peripheral blood mononuclear cells to mature osteoclasts and assessed effects of the GPR35 synthetic agonists, TCG1001 and Zaprinast, on osteoclast activity and differentiation. Both agonists stimulated significant reductions in osteoclast bone resorption and tartrate-resistant acid phosphatase (TRAP) activity, and downregulated expression of *MMP9,* a gene that regulates osteoclast bone resorption. These effects were prevented by pre-incubation of cells with a GPR35-specific antagonist. To understand GPR35 signaling pathways, we measured the phosphorylation of secondary messengers known to have important roles in osteoclast activity using AlphaLISA assays. Upon GPR35 stimulation, we observed reduced phosphorylation of c-Src, which stimulates actin ring formation necessary for bone resorption, and decreased phosphorylation of Akt, cyclic AMP (cAMP) response element-binding protein, and nuclear factor κB that drive transcription of genes required for bone resorption. Additionally, we used chemical inhibitors and siRNA knockdown to show that GPR35 couples to Gi/o and G12/13 to stimulate these signaling pathways. Finally, we compared the ability of GPR35 agonists to suppress osteoclast activity to that of current osteoporosis drugs, denosumab and alendronic acid, and showed TRAP activity was similarly suppressed under all conditions. Our findings demonstrate that GPR35 has an important inhibitory role in human osteoclast activity and have defined the signaling pathways that drive these processes. GPR35 represents a promising novel target to reduce osteoclast activity that could be exploited for osteoporosis treatments.

## Introduction

Current treatments for osteoporosis focus on prevention of bone loss or increases in bone mass using anti-resorptive, anabolic or combined anti-resorptive and anabolic drugs including bisphosphonates, parathyroid hormone (PTH)-related analogs, and sclerostin inhibitors.^1^ Although these drugs are effective in lowering fracture risk and increasing bone mass they have disadvantages including acute reactions, osteonecrosis of the jaw and atypical femur fractures for bisphosphonates,^2^ serious cardiovascular events for the anti-sclerostin drug Romosozumab, hypercalcemia for the PTH-related analog teriparatide,^3^ and rapid bone loss and increased fracture risk following cessation of treatment for denosumab.^4^ These adverse effects indicate that additional osteoporosis therapies, which could be used alone or as part of a sequential therapy approach, are required to improve patient outcomes for osteoporosis.

G protein-coupled receptors (GPCRs) are cell surface receptors that are the target of approximately one-third of all drugs approved by the US Food and Drug Administration (FDA),^5^ including teriparatide and abaloparatide that target the GPCR PTH type-1 receptor (PTH1R) to treat osteoporosis. G protein-coupled receptors with high expression in bone cells likely have an important role in osteoclast and/or osteoblast activity and could represent new targets for osteoporosis treatments. Our previous studies of gene expression in primary human osteoclasts using RNA-sequencing revealed that 144 GPCRs are expressed in osteoclasts,^6^ and subsequent investigation of a subset of these GPCRs identified four receptors that reduce the activity of primary human osteoclasts.^6^ Activation of one receptor, G protein-coupled receptor 35 (GPR35), significantly reduced nuclear translocation of the transcription factor nuclear factor of activated T cells-1 (NFATc1), which is essential for osteoclast differentiation and resorptive activity, and reduced bone resorption and tartrate-resistant acid phosphatase (TRAP) activity in mature human osteoclasts.^7^ However, the mechanisms by which GPR35 mediates these effects in human osteoclasts remain unknown.

G protein-coupled receptor 35 is officially designated an orphan receptor, as the nature of its endogenous ligand(s) remains under investigation.^8^ Several endogenous ligands (eg, the tryptophan metabolite kynurenic acid^9^ and the serotonin metabolite 5-hydroxyindoleacetic acid (5-HIAA)^10^) can activate the receptor, although they have low potency in humans^8^ or require independent verification. Therefore, studies of the physiological function of GPR35 have largely been performed with synthetic agonists such as Zaprinast or TC-G 1001,^8^^,^^11^ while antagonists such as ML145^12^ have helped confirm GPR35-specificity. These studies have revealed expression of GPR35 in immune cells, where it has a role in leucocyte and neutrophil recruitment to inflammatory sites,^10^^,^^13^ the gastrointestinal tract, where it may have a role in lipid metabolism and adipose tissue thermogenesis,^14^ and in dorsal root ganglia, where an anti-nociceptive role has been described.^15^^,^^16^ GPR35 is expressed in both osteoblasts^17^ and osteoclasts^6^ and gene expression has been shown to be downregulated in humans and mice with osteoporosis.^17^  *Gpr35^−/−^* mice have reduced bone mass due at least in part to impaired osteoblast development by reducing β-catenin activity, while activation of GPR35 in osteoporotic mice improves bone density.^17^^,^^18^ Osteoclasts were not examined in these studies. Our recent studies suggest GPR35 stimulation impairs osteoclast activity,^6^ however, we used Zaprinast to activate the receptor, which has been reported to inhibit phosphodiesterase (PDE)-5 and PDE6 at similar potencies to that at which it activates GPR35.^19^ Therefore, confirmatory studies with other receptor-specific agonists, antagonists and/or gene knock-down are required to examine the mechanisms by which GPR35 regulates osteoclast activity.

Following activation, GPCRs undergo conformational changes that mediate signaling by their associated heterotrimeric Gα/β/γ proteins. Gα consists of four subfamilies: Gs that activates cAMP, Gi/o that inhibits cAMP and is pertussis toxin (PTx) sensitive, Gq/11 that stimulates inositol trisphosphate (IP_3_) and intracellular calcium pathways, and G12/13 that couples to RhoA signaling. Studies of cell-lines (eg, HEK293) in which GPR35 is overexpressed indicate that signaling is primarily by Gi/o and G12/13^19–22^ and the reported CryoEM structure of GPR35 is with a chimeric G protein that has most similarity with G13.^23^ However, it remains uncertain which Gα protein subtypes GPR35 couples to in physiologically relevant cells, as this has largely been unexplored.

Here, we investigated the role of GPR35 in primary human osteoclasts using 2 receptor agonists (TC-G 1001 and Zaprinast), a receptor antagonist (ML145), and siRNA knockdown of GPR35. We characterized the signaling pathways activated by the receptor and activity in osteoclast monocultures and osteoclast-osteoblast co-cultures. Finally, we assessed how GPR35-induced reductions in osteoclast activity compare to two existing osteoclast-targeted osteoporosis drugs.

## Materials and methods

### Compounds

**Figure 1:**
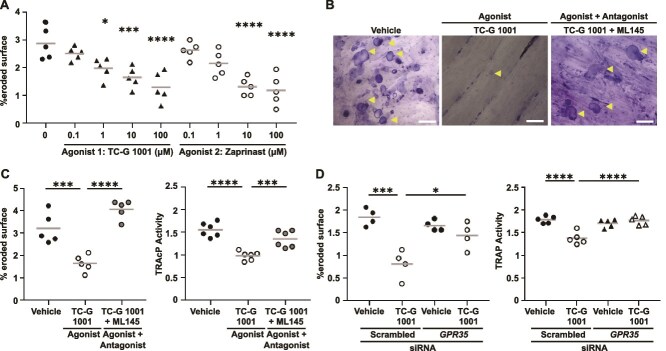
Activation of G protein-coupled receptor 35 (GPR35) reduces human osteoclast resorption. (A) Quantification of toluidine blue-stained resorption pits and trenches formed on bone slices by mature osteoclasts exposed to vehicle or four concentrations of either the TC-G 1001 or Zaprinast agonist for 3 d. (B) Representative images of resorption pits and trenches formed on bone slices by mature osteoclasts exposed to vehicle, TC-G 1001 or TC-G 1001 and ML145 antagonist. Areas of resorption are indicated by yellow arrows. Scale bar, 100 μm. (C) Quantification of bone resorption and tartrate-resistant acid phosphatase (TRAP) enzyme activity in conditioned media from osteoclasts treated with GPR35 agonist with and without antagonist, ML145. (D) Quantification of resorption pits and trenches formed on bone slices and TRAP enzyme activity by mature osteoclasts transfected with scrambled or *GPR35* siRNA and exposed to vehicle or *GPR35* agonist, TC-G 1001. Each point represents an independent donor. The gray line denotes mean in panels A, C, and D. Statistical analyses were performed by one-way analysis of variance (ANOVA) with Holm-Šídák’s multiple comparisons test. ^****^p < .0001, ^***^p < .001, ^**^p < .01.

Compounds were used at the following concentrations: Alendronic acid (10 μM, Cambridge Bioscience, Cambridge, United Kingdom), Denosumab (10 μM, Cambridge Bioscience, Cambridge, United Kingdom), GIP (10 nM, Bio-Techne, Abingdon, United Kingdom), GIP (3-30)NH2 (10 μM, Caslo, Lyngby, Denmark), ML145 (10 μM, Tocris, Abingdon, United Kingdom), PTx (300 ng/mL, Tocris), TC-G 1001 (Tocris) at 10 μM for most experiments, and a concentration range in Figure 1, TUG891 (10 μM, Tocris), YM-254890 (10 μM, Cambridge Bioscience), Zaprinast (Merck, Gillingham, United Kingdom) at 10 μM for most experiments, and a concentration range in Figure 1.

### Cell culture

All cells were maintained at 37 °C and 5% CO_2_. Primary human osteoclasts were differentiated from CD14+ monocytes, isolated from leucocyte cones obtained from anonymous blood donations from the NHS Blood and Transplant service. Approval for isolation of monocytes from human peripheral blood mononuclear cells (PBMCs) and their differentiation into osteoclasts was obtained from the local ethics committee in the United Kingdom (REC: 23/WA/0063, IRAS Project ID: 321094). Monocytes were enriched using the RosetteSep Human Monocyte Enrichment Cocktail (StemCell Technologies, Cambridge, United Kingdom) according to manufacturer’s recommendations and separated on a Ficoll-Paque gradient (VWR, Lutterworth, United Kingdom), as previously described.^24^ Monocytes were seeded in α-minimal essential medium (αMEM, Gibco, Paisley, United Kingdom) supplemented with 10% newborn calf serum (NBCS; Gibco), 1% penicillin/ streptomycin (Fisher Scientific, Loughborough, United Kingdom), and 25 ng/mL macrophage colony-stimulating factor (M-CSF) (Bio-Techne, Abingdon, United Kingdom). Monocytes were differentiated into primary human osteoclasts over 10 d. Cells were initially stimulated with M-CSF, with media refreshed every 2-3 d. Receptor activator of nuclear factor-κβ (RANKL, 25 ng/mL, Bio-Techne) was added to cells on day 8 of differentiation to stimulate osteoclast formation. Differentiation was validated microscopically on day 10, observing the presence of multinucleated cells.

Osteoblast-like cells were differentiated from the osteoblast precursor cell line, human mesenchymal stem cell with TERT immortalization (hMSC-TERT),^25^ in Dulbecco's modified Eagle medium (DMEM) supplemented with 10% NBCS, 1% penicillin/streptomycin (Gibco), 10 mM β-glycerophosphate (Sigma-Aldrich, Gillingham, United Kingdom), 10 nM dexamethasone (Sigma-Aldrich), 50 μg/mL ascorbic acid (Merck), and 2 mM L-glutamine (Gibco). For differentiation, media was replaced every 2-3 d and differentiation was complete on day 8, with alkaline phosphatase (ALP) activity assays performed to confirm differentiation. hMSC-TERT were routinely tested to ensure they were mycoplasma-free using the TransDetect Luciferase Mycoplasma Detection kit (CliniSciences Ltd., Slough, United Kingdom).

### Transfection with siRNA

Trilencer-27 siRNAs were obtained from OriGene Technologies (Herford, Germany) targeting 3 27-mer duplexes of *GNAQ* (Catalog. No. SR301847), *GNA11* (Catalog. No. SR301839), *GNA12* (Catalog. No. SR320315), *GNA13* (Catalog. No. SR307281) and *GPR35* (Catalog. No. SR301916), as well as scrambled negative controls (Catalog. No. SR30004). Primary human osteoclasts were transiently transfected with 3 siRNA oligos for each gene at 10 nM each, using the GenMute transfection reagent (SignaGen, Peterborough, United Kingdom). Cells were incubated with the transfection mix for 72 h.

### Osteoclast resorption assays

On day 10 of differentiation, all cells were collected using Accutase (Sigma, Gillingham, United Kingdom) and cell scraping and seeded on bovine cortical bone slices (Boneslices.com, Jelling, Denmark) in 96-well plates and allowed to settle for 1 h. In co-culture studies, 50 000 osteoclasts per well were seeded in RANKL-free media and left to settle before adding 12 500 osteoblast-like cells per well on top. G protein-coupled receptor agonists and/or antagonists were added, and cells were incubated for 72 h. Experiments were terminated by removal of media and the addition of dH_2_O to each well. Cells were removed from bone slices with a cotton swab, and the bone slices were stained with toluidine blue solution (1% toluidine blue, 1% sodium borate in dH_2_O, both from Sigma-Aldrich) for 20 s. To visualize and quantify areas of bone resorption, blinded microscopical analyses were performed at 10x magnification on an Olympus BX53 microscope (Olympus, Tokyo, Japan), using a 10 × 10 counting grid (24.5 mm, Graticules Optics Ltd., Tonbridge, United Kingdom) as described.^26^ Resorption was measured in 8 fields of view spanning the bone slice and represented as the percentage eroded surface per bone slice. Sample images were taken using Olympus cellSens software (Olympus).

### Tartrate-resistant acid phosphatase 5b activity assays

On day 10 of differentiation, all cells were collected using Accutase and cell scraping, then seeded in 96-well plates and exposed to agonists and/or antagonists for 72 h. Conditioned media was collected and transferred in 10 μL duplicates into a 96-well clear plate, then 90 μL TRAP solution buffer (1 M acetate (Merck), 0.5% Triton X-100 (Sigma-Aldrich), 1 M NaCl (Sigma-Aldrich), 10 mM EDTA (Fisher Scientific), 50 mM L-Ascorbic acid (Merck), 0.2 M disodium tartrate (Sigma-Aldrich), 82 mM 4-nitrolphenylphosphate (Sigma-Aldrich)) added to each well, prior to incubation in the dark for 30 min at 37 °C. The reaction was stopped by adding 0.3 M NaOH (Honeywell, SLS, Hessle, United Kingdom) and absorbance was measured at 405 nm on a SpectraMax ABS (Molecular Devices) or GloMax plate reader.

### AlphaLISA phosphorylation assays

Mature osteoclasts were plated in 96-well plates at a density of 50 000 cells per well in osteoclast monocultures and co-cultures. In co-culture studies, 50 000 osteoclasts per well were seeded in RANKL-free media and left to settle before adding 12 500 osteoblast-like cells per well on top. Cells were pre-incubated with vehicle or GPR35 antagonist for 30 min and then exposed to vehicle or GPR35 agonist for 30 min. For G_q_ protein inhibition experiments, 10 μM YM-254890 was added to cells for 30 min with vehicle or antagonist treatments. For G_i/o_ protein inhibition experiments, cells were seeded on day 9 of differentiation and exposed to 300 ng/mL PTx for 16 h prior to agonist and antagonist treatments. For siRNA studies, cells were transfected on day 10, then incubated for 72 h before AlphaLISA assays were performed.

For all assays, cells were lysed in the supplied AlphaLISA buffer (PerkinElmer, Beaconsfield, United Kingdom). Lysates were transferred to white 384-well plates (OptiPlates, PerkinElmer) in duplicates and assays performed according to manufacturer’s instructions. AlphaLISA readings were made on a PHERAstar FS (BMG Labtech, Aylesbury, United Kingdom) plate reader, and values for phosphorylated proteins normalized to Glyceraldehyde 3-phosphate dehydrogenase (GAPDH) values. The following AlphaLISA assay kits (PerkinElmer) were performed according to manufacturer’s instructions: phosphorylated forms of Akt1/2/3 (Ser473), cAMP response element-binding protein (CREB) (Ser133), c-Src (Tyr419), NFkB p65 subunit (Ser536), and p38 (Thr180/Thr182) and non-phosphorylated GAPDH.

### LANCE cAMP assays

cAMP levels was assessed using Lance Ultra cAMP assays (Revvity, Pontyclun, United Kingdom) On day 10 of differentiation all cells were collected using Accutase and cell scraping, then cells were seeded in clear 96-well plates, at a density of 50 000 cells per well, in stimulation buffer (1x Hanks Buffered Saline Solution (HBSS; Merck), 0.1% bovine serum albumin (BSA) (Sigma-Aldrich), 0.1% 3-isobutyl-1-methylxanthine (IBMX; Merck), 0.5 mM HEPES (4-(2-hydroxyethyl)-1-piperazineethanesulfonic acid) (Fisher Scientific)). Agonists and antagonists were diluted in stimulation buffer, then added to cells and incubated for 30 min at 37 °C. For G_i/o_ protein inhibition experiments, cells were seeded on day 9 of differentiation and exposed to PTx for 16 h prior to agonist and antagonist treatments. Lance cAMP assays were then performed according to manufacturer’s instructions in 96-well plates. Lysates were transferred to a white 384-well plate in duplicates and Homogeneous Time Resolved Fluorescence (HTRF) readings were made at 665 nm and 615 nm on a PHERAstar FS plate reader. Data was expressed as the HTRF ratio (665 nm/615 nm), values normalized to the vehicle controls.

### qPCR

To assess siRNA knockdown efficiency, mature osteoclasts were plated at 1 000 000 cells per well in a 6-well plate on day 10 of differentiation. For differentiation studies, isolated monocytes were seeded at 1 000 000 cells/well in a 35 mm dish. Osteoclasts were plated into media with compounds, and media with compounds were refreshed daily, with RANKL added into the media starting on day 8. RNA was extracted on days 3, 6, and 10 of osteoclast differentiation.

For all experiments, total RNA was extracted using an RNeasy Mini Kit (Qiagen, Manchester, United Kingdom). cDNA synthesis was performed using the QuantiTect Reverse Transcription Kit (Qiagen), and qPCR performed using QuantiTect primers obtained from Qiagen (Table S1). Expression was normalized to the geometric mean of 3 housekeeper genes (β-actin (*ACTB*), ribosomal protein lateral stalk subunit P0 (*RPLP0*), and ubiquitin C (*UBC*)). Threshold cycle (C_T_) values were obtained from the start of the log phase on ThermoFisher Connect software, and C_T_ values analyzed in Microsoft Excel using the Pfaffl method,^27^ then data was normalized to vehicle-treated values expressed as 1.

### IP-one assays

IP-one G_q_ assays (Revvity) were used to measure inositol phosphate-1, a stable downstream metabolite of inositol phosphate 3 (IP-3). Assays were performed as described.^28^ On day 10 of differentiation, all cells were collected using Accutase and cell scraping, and cells were seeded in clear 96-well plates, at 75 000 cells per well in 1x stimulation buffer (Revvity). Compounds were diluted in 1x stimulation buffer and incubated with cells for 30 min at 37 °C. For G_q_ protein inhibition experiments, YM-254890 was added to cells for 30 min with vehicle or antagonist treatments. IP-1-d2 and Anti-IP-1 Cryptate solutions (in lysis buffer) were added and incubated for 1 h each at room temperature. Assays were performed in duplicates with 20 μL of the mix from 96-well plates transferred to white 384-well OptiPlates, and the HTRF signal was read on a PHERAstar FS plate reader. Data was expressed as a ratio of 665 nm/620 nm, then normalized to vehicle.

### ALP activity

Alkaline phosphatase activity was measured to confirm the presence of fully differentiated osteoblast-like cells. Fully differentiated and non-differentiated hMSC-TERT cells were seeded at 20 000 cells per well in clear 96-well plates and left to settle for 48 h. After 48 h, media was removed, and cells were washed with PBS then incubated with 200 μL reaction buffer (0.06 M Na_2_CO_3_ (Sigma-Aldrich), 0.04 M NaHCO_3_ (Merck), 0.1% Triton X-100 (Sigma-Aldrich), 2 mM MgSO_4_ (Fisher Scientific), 6 mM 4-NPP (Sigma-Aldrich), in water) per well for 30 min to1 h at 37 °C. To terminate the experiment, 100 μL 1 M NaOH was added, then absorbance measured at 405 nm using a SpectraMax ABS (Molecular Devices) plate reader.

### Caspase-3/7 and senescence assays

On day 10 of differentiation, all cells were collected using Accutase and cell scraping and seeded on bovine cortical bone slices in 96-well plates. Cells were exposed to agonists for 72 h, then equal volumes of fresh media and Caspase-Glo or Beta-Glo reagent (Promega) added to each well. Wells with media only were used as background controls, and data was normalized to these values. Plates were read on a Glomax plate reader following a 30-min incubation.

### Statistical analysis

The number of experimental replicates denoted by *n* is indicated in figure legends. Data was exported to Microsoft Excel, and statistical analyses carried out using GraphPad Prism 9. Data is presented as mean ± SEM unless otherwise stated. Normality tests (Shapiro-Wilk or D’Agostino-Pearson) were performed on all datasets to determine whether parametric or non-parametric statistical tests were appropriate. A *p*-value of < .05 was considered statistically significant. Statistical analyses were performed as described in figure legends.

## Results

### Activation of GPR35 reduces human osteoclast resorption

Our previous studies have shown that activation of GPR35 with Zaprinast reduces osteoclast resorptive activity and osteoclast number.^6^ We confirmed that the activation of GPR35 reduces osteoclast resorption in a concentration-dependent manner with 2 agonists, TC-G 1001 and Zaprinast (Figure 1A), and that co-treatment with the ML145 GPR35 antagonist prevented these reductions in activity (Figure 1B-C). Activation of GPR35 also reduced TRAP activity in mature osteoclasts (Figure 1C). To assess whether the TC-G 1001 agonist specifically activated GPR35, bone resorption assays and TRAP activity were repeated in the presence of a *GPR35* siRNA and compared to a scrambled siRNA. We first confirmed that the siRNA could significantly reduce *GPR35* gene expression (Figure S1A), then assessed its effects on osteoclast activity. In the presence of scrambled siRNA, TC-G 1001 significantly reduced bone resorption and TRAP activity, which was not observed in cells transfected with *GPR35* siRNA (Figure 1D). Thus, GPR35 stimulation reduces osteoclast resorption, likely by reducing cell number.

Although we have previously shown that GPR35 stimulation reduces cell viability^7^ we did not determine the effect of receptor activation on apoptosis and cell senescence. To assess whether TC-G 1001 and Zaprinast affect apoptosis, we performed Caspase-Glo assays on mature osteoclasts exposed to either agonist for 72 h. This showed both compounds significantly enhanced apoptosis compared with cells exposed to vehicle control (Figure S2A). In contrast, GPR35 activation did not affect osteoclast senescence (Figure S2B). Thus, GPR35 reduces human osteoclast resorption at least in part by enhancing cell apoptosis.

### GPR35 activation reduces c-Src and PI3K-Akt signaling pathways in mature osteoclasts

**Figure 2:**
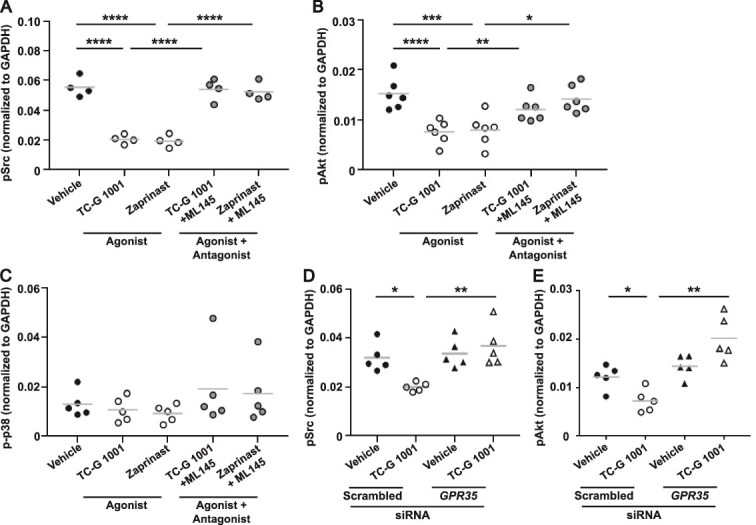
G protein-coupled receptor 35 (GPR35) activation reduces c-Src and PI3K-Akt signaling pathways in mature osteoclasts. Quantification of phosphorylated (A) c-Src (pSrc), (B) Akt1/2/3 (pAkt), and (C) p38 (p-p38) measured by AlphaLISA in osteoclasts exposed to vehicle or GPR35 agonists with or without GPR35 antagonist ML145. (D-E) Quantification of (D) c-Src and (E) Akt1/2/3 phosphorylation in mature osteoclasts transfected with scrambled or *GPR35* siRNA exposed to vehicle or *GPR35* agonist, TC-G 1001. Data in all panels was normalized to GAPDH as a housekeeper control. Each point represents an independent donor in all panels. The gray line denotes mean in panels a, B, D and E and median in panel C. Statistical analyses were performed by one-way ANOVA with Holm-Šídák’s multiple comparisons test in panels A, B, D, and E and Kruskal–Wallis with Dunn’s multiple comparisons test in C. ^****^p < .0001, ^***^p < .001, ^**^p < .01, ^*^p < .05.

We next sought to determine which signaling pathways GPR35 activates to mediate these anti-resorptive effects. The phosphorylation of three proteins, c-Src, Akt1/2/3, and p38, which have important roles in osteoclast activity and/or differentiation^29–32^ was first investigated. The phosphorylation of c-Src is important for actin ring formation in osteoclastic bone resorption.^29^ Activation of GPR35 by both TC-G 1001 and Zaprinast reduced phosphorylation of the Tyr419 c-Src residue, when compared to mature osteoclasts exposed to either vehicle or antagonist (Figure 2A), consistent with reduced bone resorption by GPR35. The phosphorylation of Akt (pAkt) can promote osteoclast resorption or osteoclast differentiation.^30^^,^^33^ Phosphorylation of Akt1/2/3 was significantly reduced in mature osteoclasts exposed to GPR35 agonists, with effects abolished in cells pre-treated with ML145 (Figure 2B). Osteoclast differentiation can also be induced by the p38 mitogen-activated protein kinase (MAPK) pathway.^31^^,^^32^ However, GPR35 activation did not affect phosphorylation of p38 (Figure 2C). Pre-treatment of osteoclasts with the *GPR35* siRNA prevented the reduction in phosphorylated c-Src (pSrc) and Akt1/2/3, further demonstrating that these responses are GPR35 mediated (Figure 2D-E).

### GPR35 activation reduces transcription factor activation and osteoclast-specific gene expression

**Figure 3:**
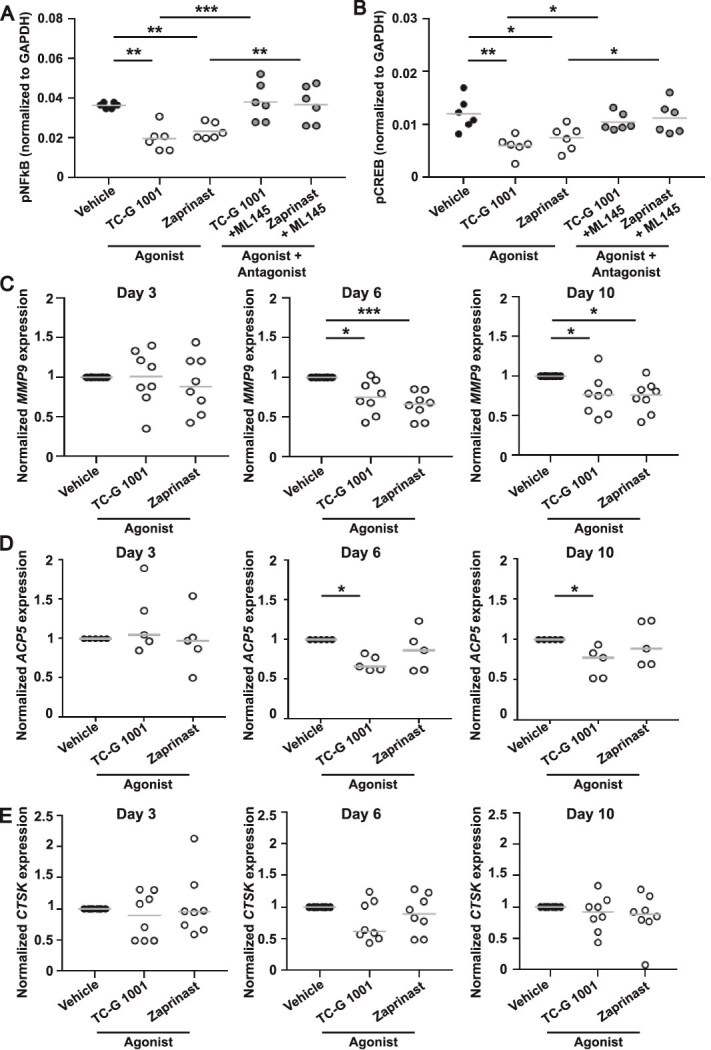
G protein-coupled receptor 35 (GPR35) activation reduces nuclear factor κB (NFκB) and cAMP response element-binding protein (CREB) signaling and suppresses *MMP9* expression. Quantification of (A) phosphorylated NFκB or (B) phosphorylated CREB in osteoclasts exposed to vehicle or GPR35 agonists with or without GPR35 antagonist ML145. Gene expression of (C) *MMP9*, (D) *CTSK*, and (E) *ACP5* in primary human osteoclasts following treatment with GPR35 agonists, TC-G 1001 and Zaprinast, on day 3, day 6, and day 10 of osteoclast differentiation, determined by qPCR. Each point represents an independent donor. The gray line denotes mean in A-B and median in C-E. Statistical analyses were performed by one-way ANOVA with Holm-Šídák’s multiple comparisons test in A-B and by Kruskal–Wallis test with Dunn’s multiple comparisons test in C-E. ^****^p < .0001, ^***^p < .001, ^**^p < .01, ^*^p < .05.

A number of signaling pathways are known to be activated downstream of c-Src and PI3K-Akt, including the nuclear factor κB (NFκB)^34–36^ and CREB.^37^ Phosphorylation of the p65 NFκB subunit and CREB protein was reduced by GPR35 activation with TC-G 1001 and Zaprinast when compared to mature osteoclasts exposed to vehicle or antagonist with agonist (Figure 3A-B). NFκB and CREB activate NFATc1 nuclear translocation, which induces the expression of genes with roles in osteoclast differentiation and activation. As GPR35 activation suppresses NFκB and CREB phosphorylation, and we have previously shown GPR35 reduces NFATc1 nuclear translocation,^6^ we assessed the expression of three known NFATc1 target genes, cathepsin K (*CTSK*), acid phosphatase 5, tartrate resistant (*ACP5*), and matrix metallopeptidase 9 (*MMP9*). To assess the effect of GPR35 stimulation on differentiation, cells were exposed to GPR35 agonists from monocyte isolation on day 1, and gene expression assessed on days 3, 6, and 10 of osteoclast differentiation. *MMP9* and *ACP5* expression was significantly reduced on days 6 and 10 by GPR35 activation when compared to cells exposed to vehicle (Figure 3C-D). No difference in *CTSK* gene expression was detected (Figure 3E). Thus, GPR35 activation may impact matrix organization rather than direct effects on resorption.

### Effects of GPR35 on bone are maintained in osteoclast-osteoblast co-cultures

**Figure 4:**
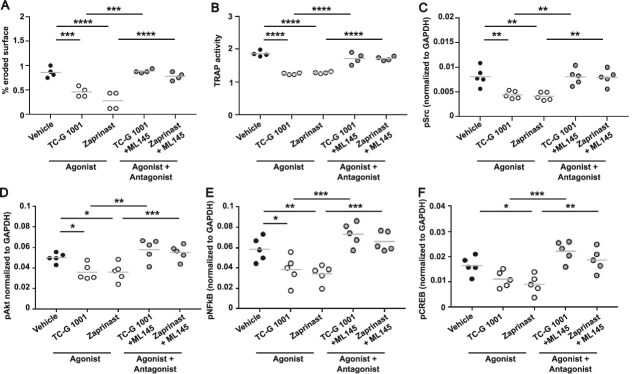
G protein-coupled receptor 35 (GPR35) mediated effects on bone resorption and signaling are maintained in osteoclast-osteoblast co-cultures. (A) Quantification of toluidine blue-stained resorption pits and trenches formed on bone slices by osteoclast-osteoblast co-cultures exposed to vehicle, TC-G 1001, or TC-G 1001 and the ML145 antagonist (B) quantification of tartrate-resistant acid phosphatase enzyme activity in conditioned media from osteoclast-osteoblast co-cultures exposed to GPR35 agonists or agonists with antagonist. (C-F) quantification of (C) phosphorylated c-Src, (D) phosphorylated Akt1/2/3, (E) phosphorylated NFκB, (F) phosphorylated cAMP response element-binding protein in osteoclast-osteoblast co-cultures exposed to GPR35 agonists or agonists with antagonist, measured by AlphaLISA. Each point represents an independent donor. The gray line denotes mean. Statistical analyses were performed by one-way ANOVA with Holm-Šídák’s multiple comparisons test for A-B and with Dunnett’s multiple comparisons test for C-F. ^****^p < .0001, ^***^p < .001, ^**^p < .01, ^*^p < .05.

In osteoclast monocultures, cells are supplemented with RANKL to induce osteoclast differentiation. To determine whether GPR35-induced reductions in osteoclast resorption are maintained in an environment in which osteoclasts respond to osteoblast-mediated RANKL secretion, the role of GPR35 was investigated in osteoclasts grown with osteoblasts differentiated from hMSC-TERT cells.^25^ The differentiation of hMSC-TERT cells to osteoblast-like cells was confirmed by measuring ALP activity (Figure S3). Mature osteoclasts were cultured with differentiated hMSC-TERT osteoblast-like cells to examine GPR35 activity in osteoclast-osteoblast co-cultures. Exposure of co-cultures to the GPR35 agonists TC-G 1001 and Zaprinast for 72 h impaired bone resorption and TRAP activity in co-cultures when compared to cells exposed to vehicle or agonist and antagonist (Figure 4A-B). GPR35 agonists also reduced pSrc, Akt1/2/3, NFkB, and CREB concentrations in osteoclast-osteoblast co-cultures, while cells with antagonist were not significantly different to cells exposed to vehicle (Figure 4C-F). Thus, GPR35-mediated effects on osteoclast activity are retained in osteoclast-osteoblast co-cultures.

### GPR35 couples to G_i/o_ and G_12/13_ proteins in primary human osteoclasts

**Figure 5:**
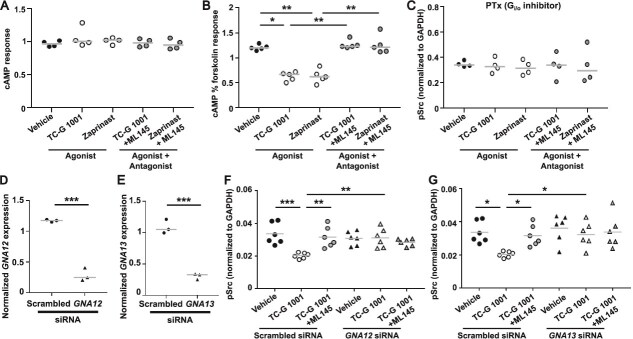
G protein-coupled receptor 35 (GPR35) couples to G_i/o_ and G_12/13_ proteins in primary human osteoclasts. (A) Quantification of cAMP levels by LANCE assays to test Gs signaling in primary human osteoclasts upon exposure to vehicle or GPR35 agonists with or without GPR35 antagonist ML145. (B) Suppression of forskolin-induced cAMP concentrations to test Gi/o signaling in osteoclasts exposed to vehicle, GPR35 agonists, or agonists with antagonist. cAMP levels were normalized to average response by donor. (C) Quantification of GPR35-mediated pSrc concentrations in osteoclasts exposed to the Gi/o inhibitor pertussis toxin. (D-E) Gene expression of (D) *GNA12* and (E) *GNA13* in mature osteoclasts exposed to scrambled or targeted siRNAs determined by qPCR. Data was normalized to the geometric mean of three housekeeper genes (*ACTB, RPLP0, UBC*). (F-G) Quantification of GPR35-mediated pSrc concentrations in osteoclasts exposed to (F) *GNA12* siRNA and (G) *GNA13* siRNA. The gray line denotes median in panels A-B and mean in panels C-G. Statistical analysis by Kruskal–Wallis test with Dunn’s multiple comparisons test in panels A-B, one-way ANOVA with Holm-Šídák’s or Dunnett’s multiple comparisons test for panels C, F, G and unpaired *t*-test in D-E. ^***^p < .001, ^**^p < .01, ^*^p < .05.

Previous studies in HEK293 and U20S cells showed overexpression of GPR35 increases signaling by G12/13 and Gi/o.^15^^,^^19–21^^,^^38^ To determine whether endogenous GPR35 functional effects in primary human osteoclasts involve these same signaling pathways, we repeated the pSrc assays in the presence of inhibitors and/or siRNAs targeting each G protein family. Signaling by cAMP was first assessed as Gs and Gi/o converge on this pathway. Mature osteoclasts were exposed to TC-G 1001 and Zaprinast and cAMP accumulation assessed by LANCE assays. There was no significant change in cAMP concentrations upon stimulation of GPR35 (Figure 5A), while increases in cAMP were detected in cells exposed to GIP, which is known to activate Gs-mediated signaling^26^ (Figure S4). Therefore, GPR35 is unlikely to signal by Gs pathways. To assess Gi/o signaling, mature osteoclasts were exposed to forskolin to elevate cAMP concentrations, then responses to GPR35 agonists and antagonists assessed. TC-G 1001 and Zaprinast significantly reduced forskolin-induced cAMP responses when compared to cells exposed to vehicle or antagonist, indicating that GPR35 signals by Gi/o pathways in primary human osteoclasts (Figure 5B). To determine whether GPR35 coupling to Gi/o contributes to signaling pathways that are known to be important for bone resorption, pSrc assays were performed in the presence of the Gi/o antagonist PTx. Pre-treatment of cells with PTx abolished GPR35-mediated effects on pSrc (Figure 5C).

To investigate G12/13 pathways, osteoclasts were transfected with siRNAs targeting either *GNA12, GNA13*, or a control siRNA. The ability of these siRNAs to reduce *GNA12* and *GNA13* gene expression was demonstrated in mature human osteoclasts by qPCR (Figure 5D-E). Assays to assess pSrc signaling with GPR35 agonists were then performed in the presence of each siRNA. Knockdown of *GNA12* and *GNA13* abolished the effects of TC-G 1001 on pSrc in mature osteoclasts (Figure 5F-G). Therefore, G12 and G13 may contribute to pSrc signaling in response to GPR35 stimulation.

The Gq/11 pathway was initially examined by measuring IP-1 concentrations in mature osteoclasts exposed to TC-G 1001 and Zaprinast. No significant differences were identified when compared to vehicle-treated cells (Figure S5A), although the FFAR4 agonist, TUG891, which we have previously shown to activate Gq/11-mediated responses,^6^ did increase IP-1 concentrations (Figure S5B). To verify these findings, we assessed GPR35-mediated effects on pSrc signaling in mature osteoclasts exposed to the Gq/11 inhibitor YM-254890 and siRNAs targeting *GNAQ* and *GNA11*. TC-G 1001 and Zaprinast retained their ability to reduce pSrc signaling in the presence of YM-254890 and the *GNAQ* and *GNA11* siRNAs, indicating that it is unlikely that GPR35 couples to Gq/11 in mature osteoclasts (Figure S5C-E).

### GPR35 agonists perform as well as current osteoporosis drugs in vitro

**Figure 6:**
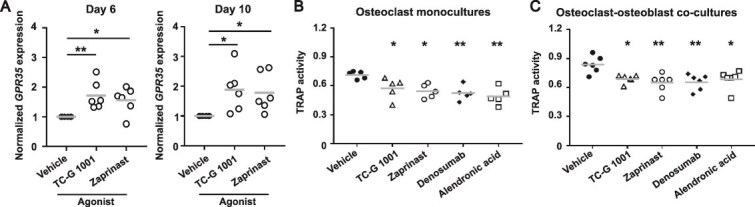
Stimulation of G protein-coupled receptor 35 (GPR35) reduces osteoclast activity similarly to current osteoporosis drugs. (A) Gene expression of *GPR35* in primary human osteoclasts following treatment with GPR35 agonists, TC-G 1001 and Zaprinast, on day 6 and day 10 of osteoclast differentiation determined by qPCR. Data was normalized to the geometric mean of three housekeeper genes (*ACTB, RPLP0, UBC*). (B-C) Quantification of tartrate-resistant acid phosphatase in conditioned media from (B) osteoclast monocultures or (C) osteoclast-osteoblast co-cultures treated with GPR35 agonists, 10 μM denosumab, or 10 μM alendronic acid. Each point represents an independent donor in all panels. The gray line denotes mean. Statistical analyses were performed by one-way ANOVA with Holm-Šídák’s multiple comparisons test with comparisons to vehicle shown in B and C. ^**^p < .01, ^*^p < .05.

Previous studies have indicated that GPR35 expression is reduced in bone marrow mesenchymal stem cells of humans and mice with osteoporosis,^17^ which could limit the feasibility of targeting GPR35. However, our previous studies have shown GPR35 expression is high in monocytes^6^ and we have shown osteoclasts can respond to GPR35 agonists (Figures 1-5). To determine whether GPR35 stimulation affects *GPR35* expression, we exposed cells to Zaprinast or TC-G 1001 for 6 and 10 d of osteoclast differentiation and compared expression by qPCR relative to cells exposed to vehicle. This showed a significant increase in *GPR35* expression at both time-points (Figure 6A).

Finally, we compared the ability of the GPR35 agonists to reduce osteoclast activity to two currently available osteoporosis drugs, denosumab and alendronic acid (alendronate). TRAP activity assays were performed in osteoclast monocultures and osteoclast-osteoblast co-cultures, with cells exposed to all compounds for 72 h. GPR35 agonists reduced TRAP activity to similar levels to denosumab and alendronic acid (Figure 6B-C).

## Discussion

We have demonstrated that stimulation of GPR35 in primary human osteoclasts impairs osteoclast activity and may be a viable target in osteoporosis therapies. We provide several lines of evidence that direct stimulation of GPR35 is responsible for these effects. Firstly, we used two synthetic agonists, TC-G 1001 and Zaprinast, that have been reported to activate GPR35 in multiple studies. While previous studies indicate that Zaprinast has off-target effects on PDE-5 and -6,^39^ PDE5 and PDE6 are expressed at very low concentrations in mature osteoclasts^6^ (Figure S6), and use of a GPR35-specific antagonist in all studies increases confidence that our findings are mediated by GPR35 activity. Moreover, Zaprinast and TC-G 1001-mediated effects were abolished in cells depleted of *GPR35* by siRNA. Furthermore, our findings are consistent with animal studies that show kynurenic acid, a putative ligand for GPR35, reduces TRAP activity in femurs of ovariectomized mice.^40^ Although previous studies of *Gpr35^−/−^* mice did not investigate the effects of depletion of the gene on osteoclast activity, it is possible that loss of the anti-resorptive effects of GPR35 on osteoclasts could also contribute to the reduced bone mass observed in these animals.

Our studies provide a comprehensive assessment of the signaling pathways activated by GPR35 within physiologically relevant cells and insights into the mechanisms by which receptor stimulation impairs osteoclast activity. Thus, GPR35 impairs the phosphorylation of c-Src, which facilitates actin ring formation in osteoclastic bone resorption,^29^ reduces pAkt1/2/3, which promotes osteoclast resorption and osteoclast differentiation,^30^^,^^33^ and subsequently reduces activation and nuclear translocation of downstream transcription factors, including CREB, NFκB, and NFATc1^6^ that regulate the expression of genes that promote osteoclast differentiation and function.^30^^,^^34–37^ Consistent with these signaling pathways, we have previously shown reductions in TRAP activity and cell viability,^6^ and here show enhanced apoptosis, reduced bone resorption, and decreased expression of *MMP9*, which has an important role in osteoclast differentiation and function downstream of NFATc1.^41^ In contrast, we did not observe a change in *CTSK* expression, although kynurenic acid has been shown to reduce *CTSK* in ovariectomized mice.^40^ This may be because there is greater variation in human samples or that estrogen deficiency in ovariectomized mice influences gene expression. Our ethics approval does not allow us to determine the age or gender of the individuals who donate blood samples, and, therefore, we do not know whether sex hormones may have influenced our findings.

These studies demonstrate that GPR35 couples predominantly to G12/13 and Gi/o signaling pathways following receptor activation, consistent with findings in transfected non-osteoclast cell-lines^19–21^^,^^42^ and the reported CryoEM structure of GPR35 bound to a chimeric G13 protein.^23^ In contrast, osteoclast-GPR35 does not appear to couple to Gq/11 pathways that have been reported in one study of transfected HEK293 cells.^11^ As we did not examine constitutive activation of the receptor, we do not know whether GPR35 is capable of signaling by other pathways in osteoclasts in the ligand-free state. However, other studies suggest constitutive activity is largely mediated by G12/13.^22^^,^^42^ Few studies have examined the G protein selectivity of endogenous GPR35. Studies of GPR35-dependent chemotactic responses in B lymphoma cells and neutrophils have shown these functions are PTx sensitive^10^ and treatment of HepG2 cells (a model of hepatocytes) with the Rho-kinase inhibitor Y27632 abolished GPR35-mediated signaling.^39^ It is likely that GPR35 has the capacity to signal by both G12/13 and Gi/o, and that the tissue-specific context (eg, G protein or effector expression levels) determines which pathway is preferentially activated.

GPR35 agonists reduced TRAP activity in osteoclasts and co-cultures to similar levels to denosumab and alendronic acid, which are currently used to treat osteoporosis. Previous studies in mice showed that GPR35 activation improves bone density in osteoporosis by direct actions on osteoblasts, but neither of these studies assessed osteoclast effects.^17^^,^^18^ Therefore, GPR35 agonism could have both anti-resorptive and anabolic effects on bone and is a promising candidate for osteoporosis treatment. Although previous studies indicate that expression of the *GPR35* gene is reduced in humans and mice with osteoporosis, stimulation of monocytes with GPR35 agonists significantly increased *GPR35* expression in pre-osteoclasts and mature osteoclasts, indicating that the receptor is a viable target. Detailed studies of GPR35 agonists in comparison to current osteoporosis drugs in mice could provide further evidence for these findings, although such studies would likely need to be performed in mice with humanized *GPR35* as most GPR35 agonists have lower potency at mouse GPR35 when compared to human.^8^^,^^39^ Additional studies may also be required to establish whether GPR35 expression in osteoclasts is affected by age and to determine whether the agonist-induced increase in gene expression is retained in cells derived from individuals with osteoporosis.

G protein-coupled receptor 35 is widely expressed in immune cells, cardiomyocytes, hepatocytes, the gastrointestinal tract, and dorsal root ganglia,^10^^,^^13–16^^,^^19^^,^^43^ and off-target effects on these cells must be considered if GPR35 is to be targeted in osteoporosis. GPR35 agonism generally has beneficial effects on these tissues, including protective roles against cardiac ischemia, anti-nociception, anti-inflammatory roles in models of colitis, and prevention and reversal of lipid accumulation.^10^^,^^13–16^^,^^19^^,^^43^ It seems unlikely that these will have detrimental adverse effects, and many will be beneficial in the generally older population with osteoporosis who may be living with multiple comorbidities. However, immune responses may need to be monitored in these patients. As GPR35 agonism is increasingly investigated to treat inflammatory bowel disease, non-alcoholic fatty liver disease, and cardiac ischemia^8^^,^^39^^,^^43^ it is likely that improved highly potent small molecules will be designed to target the receptor and these compounds could have efficacy in treating osteoporosis.

Our study has several limitations, including that our findings are currently restricted to in vitro studies. Further investigation of GPR35 agonists in animal models will be required to investigate the efficacy of targeting this receptor. Additionally, most of our studies focused on mature osteoclasts as *GPR35* expression is low in early stages of differentiation (Figure S4) and it is more likely that GPR35 has its anti-resorptive role in late differentiation (*>*day 6).

In summary, we have demonstrated that GPR35 has an anti-resorptive role in human osteoclasts by reducing multiple phosphorylated proteins downstream of G12/13 and Gi/o signaling pathways. Drugs that stimulate GPR35 could have anti-resorptive and anabolic effects on bone and represent a viable new target for osteoporosis treatments.

## Supplementary Material

Supplementary_Appendix_ziaf131

## Data and materials availability

All data needed to evaluate the conclusions in the paper are present in the paper and/or the Supplementary Materials. Enhanced expression of *GPR35* in mature osteoclasts was first identified in RNA-sequencing data generated from cultured primary osteoclasts derived from eight human donors have been deposited in the Gene Expression Omnibus (GEO) database under accession code GSE246769. Processed scRNA-seq data are available at the Open Science Framework: https://osf.io/9xys4/.
