# Supplementary material for: G protein-coupled receptor 35 stimulation reduces osteoclast activity in primary human bone cells
Source: JBMR Plus. 2025 Aug 6;9(10):ziaf131. doi: 10.1093/jbmrpl/ziaf131 (PMC12445851; doi:10.1093/jbmrpl/ziaf131)
Supplement: Supplementary_Appendix_ziaf131 [file supplementary_appendix_ziaf131.docx]

## Supplementary Appendix

**

**

**Supplementary Figure 1 Validation that siRNA significantly reduces *GPR35* gene expression**

Gene expression of *GPR35* in mature osteoclasts exposed to scrambled or *GPR35-*targeted siRNAs determined by qPCR. Data was normalized to the geometric mean of three housekeeper genes (*ACTB, RPLP0, UBC*). Each point represents an independent donor. The gray line shows mean. Statistical analyses were performed by unpaired t-test. ****p<0.0001.

**
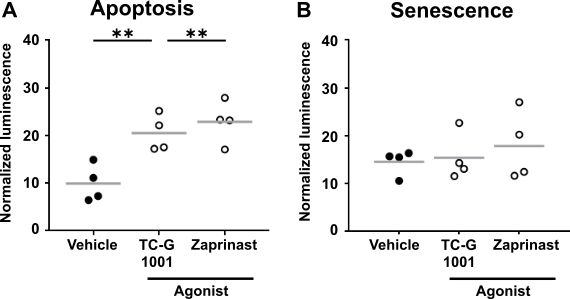
**

**Supplementary Figure 2 Activation of GPR35 enhances osteoclast apoptosis**

(**A**) Quantification of apoptosis measured by Caspase-Glo and (**B**) senescence measured by Beta-glo assays in osteoclasts exposed to vehicle or GPR35 agonists for 72 hours. Luminesence values were normalized to media only controls. Gray line denotes mean. Statistical analyses were performed by one-way ANOVA with Dunnett’s multiple comparisons test. **p<0.01.


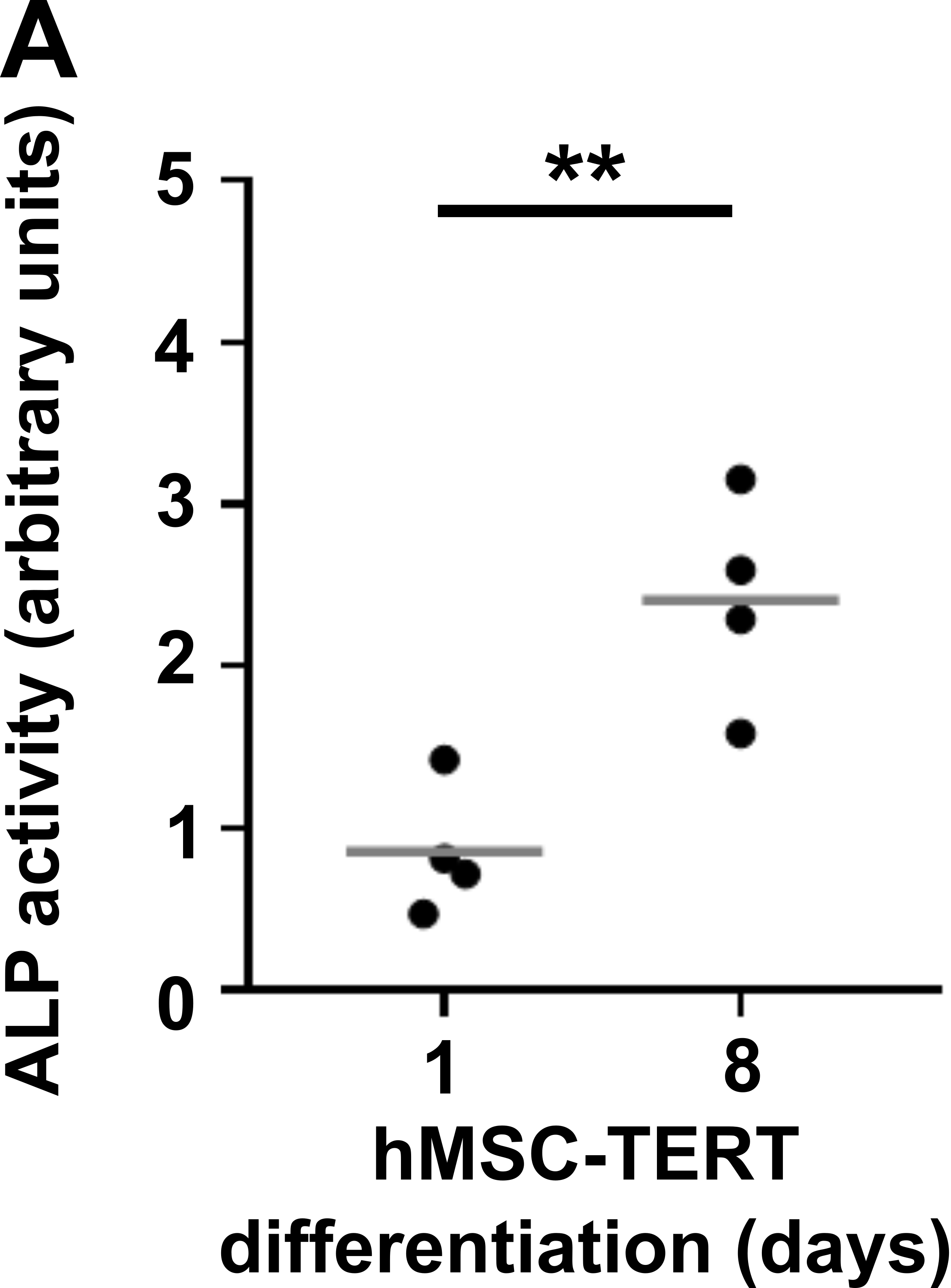


**Supplementary Figure 3 hMSC-TERT cells have increased alkaline phosphatase activity following differentiation**

Quantification of alkaline phosphatase activity in undifferentiated hMSC-TERT cells (day 1) and after eight days differentiation to osteoblasts. Each point represents an independent passage of cells. The gray line shows the mean. Statistical analysis was performed by unpaired t-test. **p<0.01.


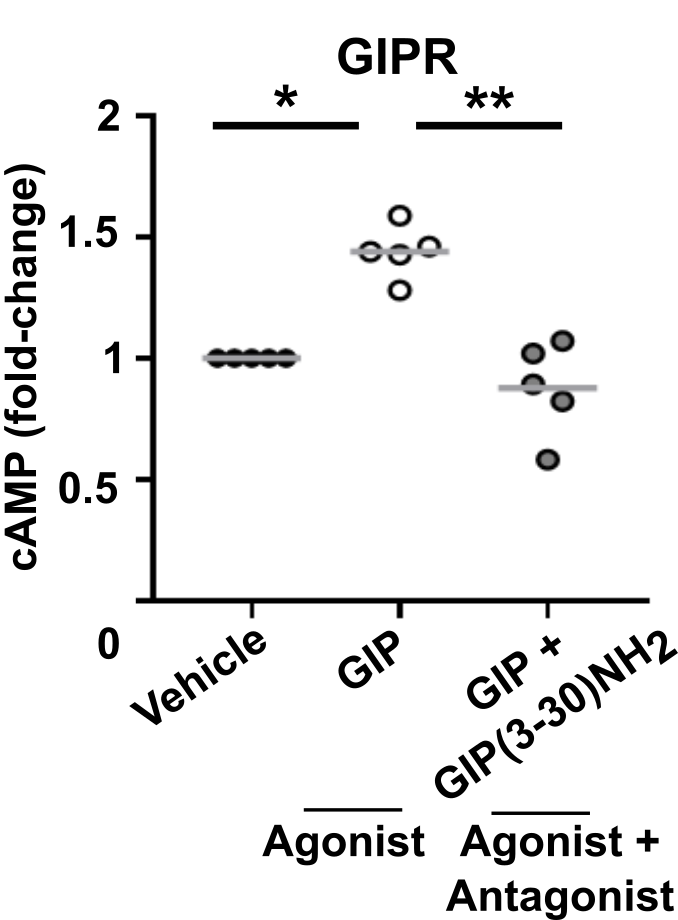


**Supplementary Figure 4 Stimulation of GIPR enhances cAMP in primary human osteoclasts**

(**A**) cAMP concentrations measured by LANCE cAMP assays in primary human osteoclasts exposed to vehicle, GIP or GIP with GIPR antagonist, GIP(3-30)NH_2_. GIPR is known to activate Gs-cAMP signaling^(^[^1^](#_ENREF_1)^)^. Each point represents an independent donor. The gray line denotes median. Statistical analysis by Kruskal-Wallis test with Dunn’s multiple comparisons testing. **p<0.01, *p<0.05.


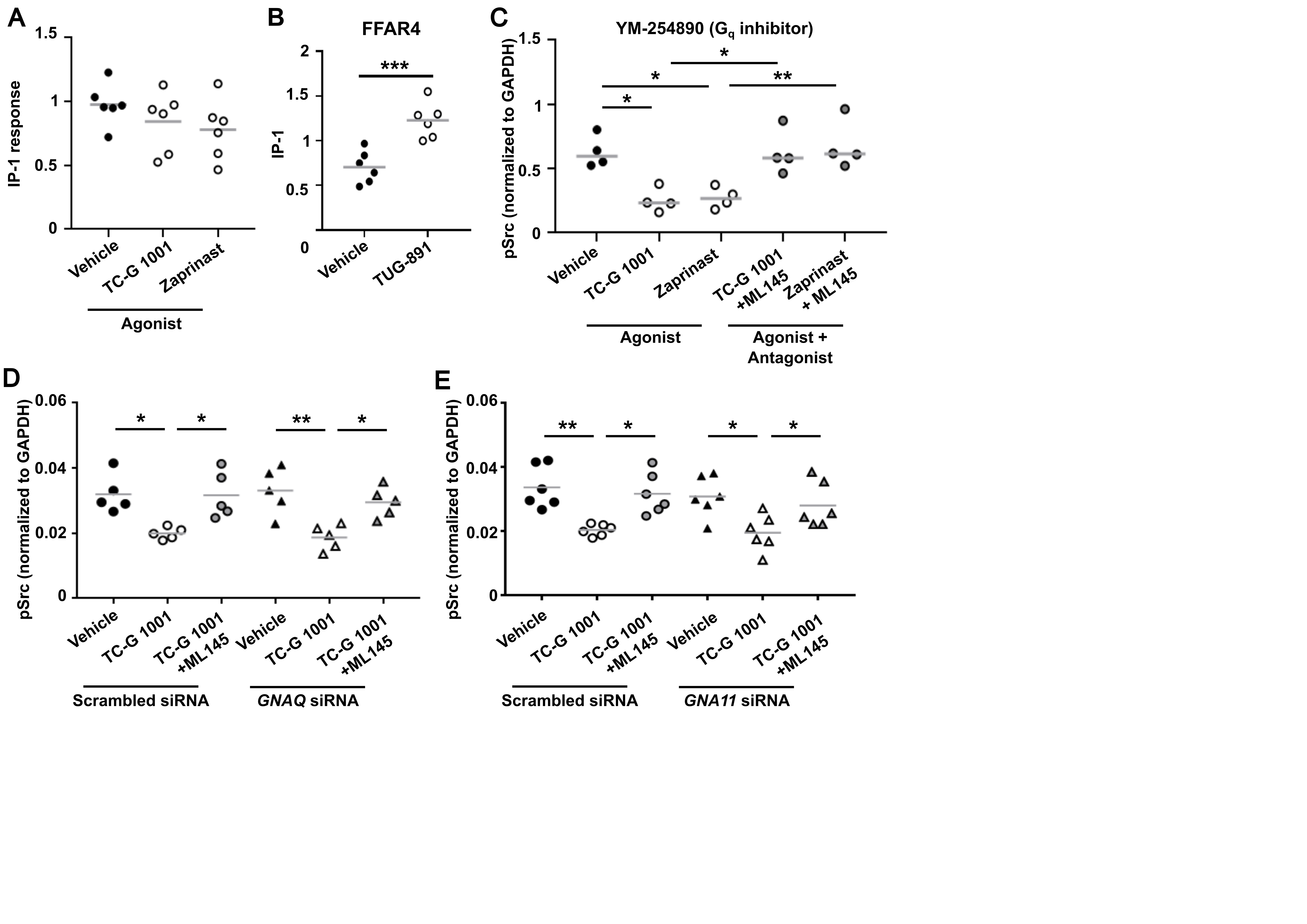


**Supplementary Figure 5 GPR35 does not couple to G_q/11_ proteins in primary human osteoclasts**

(**A**) IP-1 concentrations measured by IP-one assays in primary human osteoclasts exposed to vehicle or the GPR35 agonists, TC-G 1001 and Zaprinast. (**B**) IP-1 concentrations measured by IP-one assays in primary human osteoclasts exposed to vehicle or TUG-891, an agonist for FFAR4 which we have previously shown activates Gq signaling^(^[^2^](#_ENREF_2)^)^. (**C-E**) Quantification of GPR35-mediated pSrc concentrations in osteoclasts exposed to (C) the Gq/11 inhibitor YM-254890, (D) *GNAQ* siRNA and (E) *GNA11* siRNA and treated with agonist TC-G 1001 with or without GPR35 antagonist ML145. Each point represents an independent donor. The gray line denotes median in A and mean in other panels. Statistical analysis by Kruskal-Wallis test with Dunn’s multiple comparisons testing in A, unpaired t-test for C and one-way ANOVA with Holm-Šídák's or Dunnett’s multiple comparisons test for panels D-F. ***p<0.001, **p<0.01, *p<0.05.

**
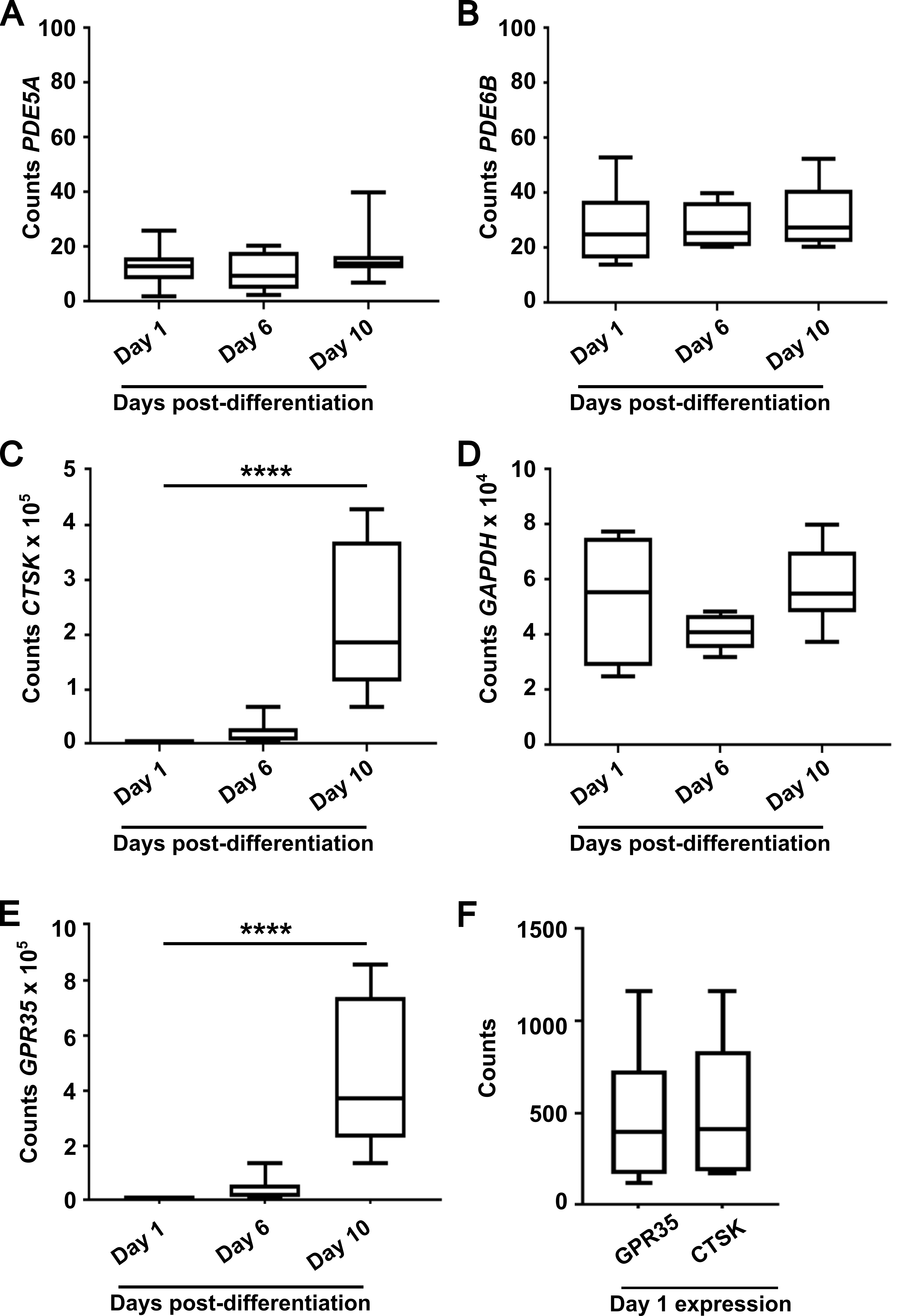
**

**Supplementary Figure 6 Expression of *PDE5A* and *PDE6B* is low in mature human osteoclasts**

Box plot of the RNA-seq based gene expression levels of: (**A**) phosphodiesterase 5A (*PDE5A*), (**B**) phosphodiesterase 6B (*PDE6B*), (**C**) glyceraldehyde-3-phosphate dehydrogenase (*GAPDH*), (**D**) cathepsin K (*CTSK*), and (**E**) *GPR35* from N=8 donors from previous studies^(^[^2^](#_ENREF_2)^)^. *CTSK* and *GPR35* expression at day 3 is 10 – 100x higher than *PDE5A* and *PDE6B*. (**F**) Counts for *CTSK* and *GPR35* at day 1 showing that expression is low before differentiation to osteoclasts. Statistical analyses were performed by one-way ANOVA with Dunnett’s multiple comparisons test. ****p<0.0001.

### Supplementary Table 1 Primers used in qPCR experiments

| **Gene Function** | **Gene name** | **Protein name** | **Gene Globe ID** |
| --- | --- | --- | --- |
| Housekeeper | *ACTB* | β-actin | QT00095431 |
|  | *RPLP0:* | Ribosomal protein lateral stalk subunit P0 | QT00075012 |
|  | *UBC* | Ubiquitin C | QT00234430 |
| Osteoclast activity | *ACP5* | Acid phosphatase 5, tartrate resistant (TRAP) | QT00199801 |
|  | *CTSK* | Cathepsin K | QT00093856 |
|  | *MMP9* | Matrix metalloproteinase 9 | QT00040040 |
| G protein activity | *GNA11* | Gα11 | QT00084987 |
|  | *GNA12* | Gα12 | QT00235858 |
|  | *GNA13* | Gα13 | QT00079968 |
|  | *GNAQ* | Gαq | QT00037296 |
| GPCR | *GPR35* | G protein-coupled receptor 35 | QT02403128 |

**References**

1. Hansen MS, Soe K, Christensen LL, Fernandez-Guerra P, Hansen NW, Wyatt RA, et al. GIP reduces osteoclast activity and improves osteoblast survival in primary human bone cells. Eur J Endocrinol. Jan 10 2023;188(1).

2. Hansen MS, Madsen K, Price M, Søe K, Omata Y, Zaiss MM, et al. Transcriptional reprogramming during human osteoclast differentiation identifies regulators of osteoclast activity. Bone Research 2024 12:1. 2024;12(1):1-19.
